# Effect of Ascorbic Acid on Differentiation, Secretome and Stemness of Stem Cells from Human Exfoliated Deciduous Tooth (SHEDs)

**DOI:** 10.3390/jpm11070589

**Published:** 2021-06-22

**Authors:** Shilpa Bhandi, Ahmed Alkahtani, Mohammed Mashyakhy, Abdulaziz S. Abumelha, Nassreen Hassan Mohammad Albar, Apathsakayan Renugalakshmi, Mazen F. Alkahtany, Ali Robaian, Asma Saleh Almeslet, Vikrant R. Patil, Saranya Varadarajan, Thodur Madapusi Balaji, Rodolfo Reda, Luca Testarelli, Shankargouda Patil

**Affiliations:** 1 Department of Restorative Dental Sciences, College of Dentistry, Jazan University, Jazan 45412, Saudi Arabia; shilpa.bhandi@gmail.com (S.B.); dr.mashyakhy@gmail.com (M.M.); nalbar01@gmail.com (N.H.M.A.); 2 Department of Restorative Dental Sciences, Division of Endodontics, College of Dentistry, King Saud University, Riyadh 11451, Saudi Arabia; ahkahtani@ksu.edu.sa (A.A.); Malkahtany@ksu.edu.sa (M.F.A.); 3 Department of Restorative Dental Sciences, College of Dentistry, King Khalid University, Abha 61421, Saudi Arabia; aabumelha@kku.edu.sa; 4 Department of Preventive Dental Sciences, Pedodontics Division, College of Dentistry, Jazan University, Jazan 45412, Saudi Arabia; arenu27@yahoo.co.in; 5 Department of Conservative Dental Sciences, College of Dentistry, Prince Sattam bin Abdulaziz University, Alkharj 11942, Saudi Arabia; ali.alQahtani@psau.edu.sa; 6 Department of Oral and Maxillofacial Surgery and Diagnostic Sciences, Riyadh Elm University, Riyadh 12611, Saudi Arabia; Asma.Almeslet@riyadh.edu.sa; 7 Biogenre Private Limited, Pune 412105, India; patilvikrant.r@gmail.com; 8 Department of Oral Pathology and Microbiology, Sri Venkateswara Dental College and Hospital, Chennai 600130, India; vsaranya87@gmail.com; 9 Department of Periodontology, Tagore Dental College and Hospital, Chennai 600127, India; tmbala81@gmail.com; 10 Department of Oral and Maxillofacial Sciences, Sapienza University of Rome, 00161 Rome, Italy; rodolforeda17@gmail.com (R.R.); luca.testarelli@uniroma1.it (L.T.); 11 Department of Maxillofacial Surgery and Diagnostic Sciences, Division of Oral Pathology, College of Dentistry, Jazan University, Jazan 45412, Saudi Arabia

**Keywords:** ascorbic acid, cytokines, differentiation, growth factors, mesenchymal stem cells, SHEDs, stemness

## Abstract

Stem cells from human exfoliated deciduous teeth (SHEDs) are considered a type of mesenchymal stem cells (MSCs) because of their unique origin from the neural crest. SHEDs can self-renewal and multi-lineage differentiation with the ability to differentiate into odontoblasts, osteoblast, chondrocytes, neuronal cells, hepatocytes, adipocytes, etc. They are emerging as an ideal source of MSCs because of their easy availability and extraordinary cell number. Ascorbic acid, or vitamin C, has many cell-based applications, such as bone regeneration, osteoblastic differentiation, or extracellular matrix production. It also impacts stem cell plasticity and the ability to sustain pluripotent activity. In this study, we evaluate the effects of ascorbic acid on stemness, paracrine secretion, and differentiation into osteoblast, chondrocytes, and adipocytes. SHEDs displayed enhanced multifaceted activity, which may have applications in regenerative therapy.

## 1. Introduction

Mesenchymal stem cells (MSCs) are pluripotent progenitor cells that undergo mitosis several times and eventually differentiate into osteoblasts, chondrocytes, and adipocytes, etc. MSCs are currently being considered for the management of various diseases [1,2,3,4]. Moreover, the secretome of mesenchymal stem cells contains several cytokines, growth factors that are responsible for the various effects of transplantation of stem cells. Although these stem cells possess regenerative potential, the complete healing of tissues can take place with the use of scaffolds along with the stem cells and their secretomes [5]. It is vital to choose suitable resorbable biomaterials as scaffolds for stem cells and possess porosity values of 90%. Among the various techniques for developing resorbable biomaterials a novel combination of thermally induced phase separation with autologous stem cells derived from a periapical cyst was reported by Tatullo et al [6] 

However, when transplanted in vivo, these cells have a low survival rate, thereby limiting their therapeutic applications. Earlier studies in regenerative medicine have considered the functions of paracrine secretion of MSCs [3,7,8]. These secretions can migrate to inflamed tissues and exert anti-inflammatory and immunosuppressive effects on both the adaptive and innate immune systems. Paracrine effects of MSCs include immunomodulation, angiogenesis, antiapoptosis, sustenance to the growth of cells, and chemo-attraction properties [9,10,11]. Transplantation of the conditioned medium (CM) that contains these paracrine factors have been reported to enhance wound healing in animal models [8,12,13]. The ease of access and growth of MSCs, along with the properties of paracrine secretion of MSCs, could make them a viable therapeutic strategy for various diseases [14,15]. Numerous studies have assessed the therapeutic effect of MSCs in cases of orthopedic damage, liver diseases, graft vs. host disease on bone marrow transplantation, autoimmune diseases, and cardiovascular diseases [16,17,18]. Overexpression of anticancer genes induced by MSCs could be a strategy for the management of cancer [19]. In vitro and in vivo studies on MSCs advance clinical research, due to the advantages, such as plasticity, stemness, and absence of adverse reactions or tumor formation upon transplantation. Among the various sources of MSCs, the dental pulp is anticipated to be an almost ideal source of multipotent MSCs, which can be utilized in areas of clinical research, including regenerative dentistry, repair of orthopedic injuries, and treatment of degenerative neural disorders [20,21]. The advantages of dental pulp stem cells include convenient isolation, minimal ethical consideration, and negligible immunogenic reactions permitting allogeneic cell transplantation [22,23,24]. New MSCs-like cell populations are being acquired from different dental and oral tissues, such as human exfoliated deciduous teeth (SHED). They are considered a valuable source for cellular banking and longstanding cryopreservation [25,26,27]. The characteristics of SHEDs include greater propagation rate, enhanced clonogenicity, and elevated osteoinductive capacity in vivo in comparison with other types of MSCs [4,28]. Studies have demonstrated that oral MSCs derived from human dental pulp exhibit in vitro pluripotency and can differentiate into several cell populations, including odontoblast, chondrocytes, osteoblasts, and neural cells. SHEDs can be cryopreserved for long periods of time without loss of cell viability. Studies examining SHED cryopreserved for two years reported similar properties, obtained from fresh tissue [9,17,29]. SHEDs show expression of definite markers that are exclusively expressed by MSCs and other pluripotent stem cells, which include CD106, STRO-1, SOX2, NANOG, and OCT4 [30,31]. Ideally, dental pulp from deciduous teeth should be obtained from healthy teeth without pathologies, such as dental caries, teeth with minimum 2/3rd root resorption, and intact 1/3rd of root to ensure vascularity sufficient to keep the cells alive, or teeth without mobility to prevent bacterial contamination. Several studies have reported the therapeutic effects of MSCs obtained from the pulp of deciduous teeth. Research has demonstrated that SHED or SHED conditioned medium reduces wrinkles caused by UV-B photo-damage when injected subcutaneously. They also resulted in increased collagen synthesis, proliferation, and migration of human dermal fibroblast [32,33,34,35]. In addition to these therapeutic effects, SHEDS have other applications in the field of Dentistry. Prasad et al. reported rapid healing of periapical tissues and root growth in teeth with open apices when stem cells were used [36]. Yang et al. reported the formation of dentin and periodontal ligament-like fibrous tissues when stem cells complexed with treated dentin matrix were transplanted subcutaneously into nude mice. Future studies could explore pulp capping in pediatric patients, and possibly tooth regeneration in congenital agenesis of teeth [37]. Nakajima et al. reported that SHED produces osteoid and a rich network of collagen fibers greater than human dental pulp stem cells (HDPSCs) and human bone marrow stem cells (HBMSCs) [38].

The higher bone regeneration ability of SHED makes it the richest source of stem cells for the reconstruction of the alveolar cleft. This regeneration ability could have applications in the management of cleft lip and cleft palate in pediatric patients. Other dental applications of SHED include managing orofacial skeletal defects, periodontitis, temporomandibular joint osteoarthritis (TMJOA), and post-traumatic lesions with transection of the facial nerve [39,40,41,42,43]. Although MSCs have several advantages, there are concerns regarding their sourcing and efficacy in therapeutic applications [44,45]. Thus, there is a need for research on properly defined culture conditions for the preservation of stemness and enhancement of the paracrine secretion of MSCs. Earlier research has demonstrated that the microenvironment of cell culture influences the secretion and differentiation potential of pulp cells [19,20]. Ascorbic acid (AsA), commonly known as Vitamin C, is an organic compound with a lactone structure. AsA has several physiological functions, such as eliminating reactive oxygen species due to oxidative stress and facilitation of mitosis that may reduce and suppress the effects of aging. Ascorbic acid has been widely used as an additive in the culture medium for cell growth and differentiation, especially on the commitment of the cell towards a lineage [9,21,31]. Considering the multifarious activity of defined culture conditions, we hypothesized that a suitable culture condition would modulate the functional activity of SHEDs in terms of differentiation potential, both qualitatively and quantitatively, and would trigger paracrine secretion. Optimal changes in the chemical and physical conditions in maintaining DPSCs are likely to augment their efficacy to treat a particular disorder. This study determines the in vitro effect of ascorbic acid (vitamin C) on the stem cell culture isolated from human exfoliated deciduous teeth pulp tissue to augment MSC culture, differentiation, and to enrich the paracrine secretion.

## 2. Materials and Methods

### 2.1. Sample Collection

The study protocol was approved by Scientific Research (IRB) CODJU-19705, College of Dentistry, Jazan University. Human deciduous teeth with incomplete root resorption were extracted under local anesthesia following aseptic protocols from healthy subjects aged 6–12 with appropriate oral hygiene (n = 5). Informed consent was obtained in accordance with institutional ethics considerations. The pulp was extracted in sterile conditions and transferred to the laboratory directly for further processing. The pulp tissue was stored in 1X phosphate-buffered saline at 4 °C and immediately processed within 2 h for cell isolation.

### 2.2. Culture and Expansion of SHEDs

Isolation and characterization of SHEDs were carried out using the explant culture method previously described [4]. Pulp tissue was minced into tiny fragments and placed in 35 mm polystyrene plastic culture dishes. Fetal Bovine Serum (FBS) (Gibco, Rockville, MD, USA) was added to the tissues to cover them completely. The samples were incubated for 24 h at 37°C and 5% CO_2_; the whole DPSCs culture system was further maintained in DMEM (Invitrogen, Carlsbad, CA, USA) supplemented with 20% FBS and antibiotic-antimycotic solution at the same temperature and CO_2_ conditions. The culture medium was replenished twice weekly, and the cell growth, health, and morphology were monitored regularly with an inverted phase-contrast microscope (Olympus CKX53, Tokyo, Japan). At 70–80% confluence, cells were detached using 0.25% Trypsin-EDTA solution (Invitrogen, Carlsbad, CA, USA) and transferred to a bigger 25-cm^2^ polystyrene culture flask (Nunc, Rochester, NY, USA). Confluent SHEDs were detached using 0.25% Trypsin-EDTA solution and then continuously passaged in for expansion and further experiments. Cells from passages 2 to 4 were used in the experimentation.

### 2.3. Characterization of SHEDs Using Flow Cytometry

For flow cytometry analysis, confluent DPSCs were harvested with trypsinization and washed with PBS twice. Cells were then incubated for 30 min at 4 °C with antihuman-CD73-APC, antihuman-CD90-APC, antihuman-CD105-APC, antihuman-CD34-PE, antihuman-CD45-FITC, and antihuman-HLA-DR-APC antibodies (Miltenyi Biotec, Auburn, CA, USA). Antibody-stained cells were washed twice with PBS, and 10,000 cells per sample were acquired on Attune NxT Flow Cytometer (Thermo Fisher Scientific, Waltham, MA, USA). Isotype control was used for detection and to differentiate between positive and negative signals.

### 2.4. 3-(4,5-Dimethylthiazol-2-yl)-2,5-diphenyltetrazolium Bromide (MTT) Assay

3-(4,5-dimethylthiazol-2-yl)-2,5-diphenyltetrazolium bromide (MTT) assay of DPSC following quercetin treatment: The cells were seeded in 96-well plates (1 × 10^4^ cells per well) and treated with increasing concentrations of L-ascorbic acid (Sigma Aldrich, St. Louis, MO, USA) (10 µM, 25 µM, 50 µM, and 100 µM). The cytotoxicity of ascorbic acid to DPSCs was measured using the MTT assay. The cells were seeded into 96-well plates and incubated with appropriate media for 24 h, 48 h, and 72 h. Following incubation, MTT solution (Sigma-Aldrich Corp., St. Louis, MO, USA) was mixed in each well at a concentration of 0.5 mg/mL. The mixing plates were further incubated for 4 h at 37 °C. Subsequently, the medium was removed, and 100 µL dimethyl sulfoxide (DMSO) (Sigma-Aldrich Corp., St. Louis, MO, USA) was added to each well. The absorbance was measured at 570 nm using a Multiskan Spectrum spectrophotometer (Thermo Scientific, San Jose, CA, USA).

### 2.5. Adipogenic Differentiation

For adipogenic differentiation, the SHEDs were seeded in a 24-well plate (2500/cm^2^) (Nunc, Rochester, NY, USA) with a complete growth medium. After 24 h, the complete growth medium was replaced with adipogenic media (DMEM supplemented with 10% FBS, 1 µM dexamethasone, 10 µM insulin, and 200 µM indomethacin. 0.5 mM isobutyl-methylxanthine (Sigma-Aldrich Corp., St. Louis, MO, USA) was introduced to the cells twice a week for three weeks. Two experimental groups were created, namely, the induction group, and 10 µM of ascorbic acid treatment with the induction group. Differentiated adipocytes were fixed with 4% paraformaldehyde and confirmed with 0.3% oil red O staining for 1 h.

### 2.6. Osteogenic Differentiation

A cell density of 2500 cells/cm^2^ was used in a 24-well plate (Nunc, Rochester, NY, USA) with a complete growth medium. After 24 h, the complete growth medium was replaced with an osteogenic induction medium of DMEM with 1% antibiotic-antimycotic, 0.1 µM of dexamethasone, 50 µM of ascorbate-2-phosphate, and 10 mM of β-glycerophosphate (Sigma-Aldrich Corp., St. Louis, MO, USA). Two experimental groups were created: The induction group, and 10 µM of ascorbic acid treatment with the induction group. The medium was replaced with a fresh induction medium of the same composition twice a week. After 21 days, cell differentiation towards osteogenic lineage was analyzed. The cells were fixed with 4% paraformaldehyde, and 2% alizarin red S (pH 4.1–4.3) staining was performed for 20 min.

### 2.7. Chondrogenic Differentiation

The cell density of 2500 cells/cm^2^ was used in a 24-well plate (Nunc, Rochester, NY, USA) with a complete growth medium. After 24 h, the complete growth medium was replaced with a chondrogenic induction medium of DMEM with 1X-ITS, 1 mM of sodium pyruvate, 100 nM of dexamethasone, 50 µg/mL of ascorbate-2-phosphate, 40 µg/mL of L-proline, and 10 ng/mL of TGF-β3 (Sigma-Aldrich Corp., St. Louis, MO, USA). Two experimental groups were created: The induction group, and 10 µM of ascorbic acid treatment with the induction group. The cultures were incubated for 28 days at 37°C in a 5% CO2 incubator. The medium was replaced every 2–3 days. After 28 days, cell differentiation towards chondrogenic lineage was analyzed using alcian blue staining. Cells were fixed with 4% paraformaldehyde and stained for glycosaminoglycans using 2% toluidine blue.

### 2.8. Cytometric Bead Array for the Detection of Cytokines and Growth Factors

A cell density of 2500 cells/cm^2^ was used in a 24-well plate (Nunc, Rochester, NY, USA) with a complete growth medium. Two experimental groups were created; the control group (without treatment) and the treated group (10 µM ascorbic acid treatment). The spent medium was collected from both groups after 48 h of incubation. A cytometric bead array was performed to determine the levels of the cytokines and growth factors in conditioned media. LEGENDplex™ Human Growth Factor Panel (Ang-2, EGF, Ang-1, FGF-basic, HGF, IGF-1, TGF-b, SCF, TGF-α, and VEGF) was used to detect growth factors. LEGENDplex™ Human Immune Panel (PGE-2, SDF-1, TGF-β1, IDO, NO, CCL2, IL-17A, IL-6, IL-10, and TNF-α) was used to detect cytokines following the manufacturer’s guidelines. 25 μL of the conditioned media was incubated with the microbeads, and detection antibodies were introduced. LEGENDplex™ Human Bone Metabolism Panel (OPG, OPN, ALPL, ACP5, Leptin, RANKL, PTH, BMP-2, DKK-1) was used according to the manufacturer’s guidelines. The samples were acquired on a flow cytometer and analyzed using LEGENDplex™ Data Analysis Software.

### 2.9. Real-Time PCR for Analysis of Gene Expression

A cell density of 2500 cells/cm^2^ was used in a 24-well plate (Nunc, Rochester, NY, USA) with a complete growth medium. Two experimental groups were created; the control group (without treatment) and the treated group (10 µM ascorbic acid treatment). The cells were incubated for 48 h. RNA extraction was done by using the Trizol method. 1 μg RNA was transcribed reversely by using a cDNA synthesis kit following the manufacturer’s guidelines. Quantitative analysis of genes of interest was done by using SYBR Green PCR master mix on a Real-Time PCR machine. Normalization of expressions of target genes to ß-actin was done by using the ΔΔCt technique. The quantification of data was done by using the 2^–ΔΔCt^ technique and shown as normalized relative gene expression to that of the average CT for the β-actin gene. The primers are listed in Table 1.

## 3. Results

### 3.1. Morphological Characteristics

**Figure 1:**
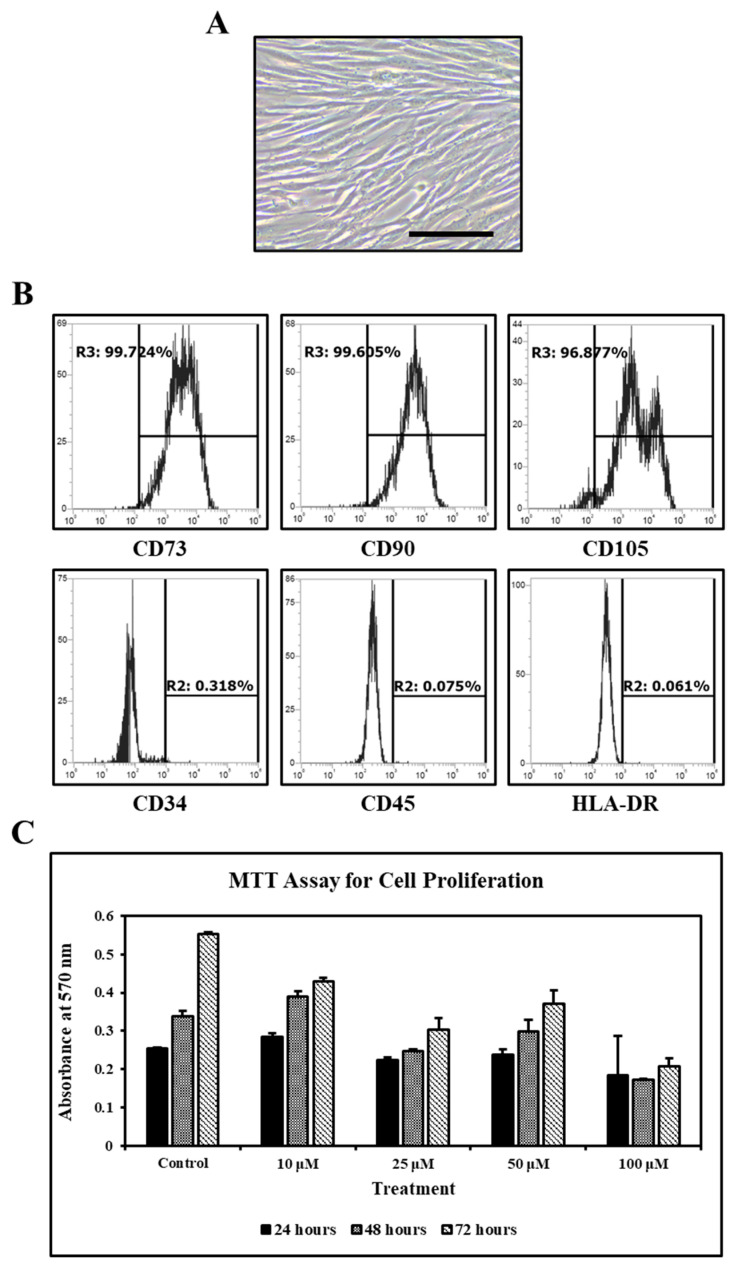
Isolation and characterization of SHEDs and MTT assay. (**A**). SHEDs at Passage 2. (**B**). Flow Cytometry analysis of SHEDs for MSC-specific cell surface markers (n = 5). Scale bar = 100 µm. (**C**)**.** MTT assay for the proliferation of SHEDs after ascorbic acid treatment.

The morphological features of SHEDs isolated by explant culture technique were monitored with a microscope. SHEDs showed elongated spindle-shaped morphology (Figure 1A).

### 3.2. MSC Specific Cluster of Differentiation Markers

SHEDs expressed CD73, CD90 and CD105 and were negative for CD34 and CD45 and HLA-DR (MHC class-II cell surface receptor) (Figure 1B).

### 3.3. Effect of Ascorbic Acid on the Viability of SHEDs

Ascorbic acid in concentrations of 10 µM, 25 µM, and 50 µM did not affect the viability of SHEDs. However, ascorbic acid at a concentration of 100 µM showed reduced viability of SHEDs (Figure 1C).

### 3.4. Ascorbic Acid Treatment Enhances Osteogenic and Chondrogenic Differentiation Potential

**Figure 2:**
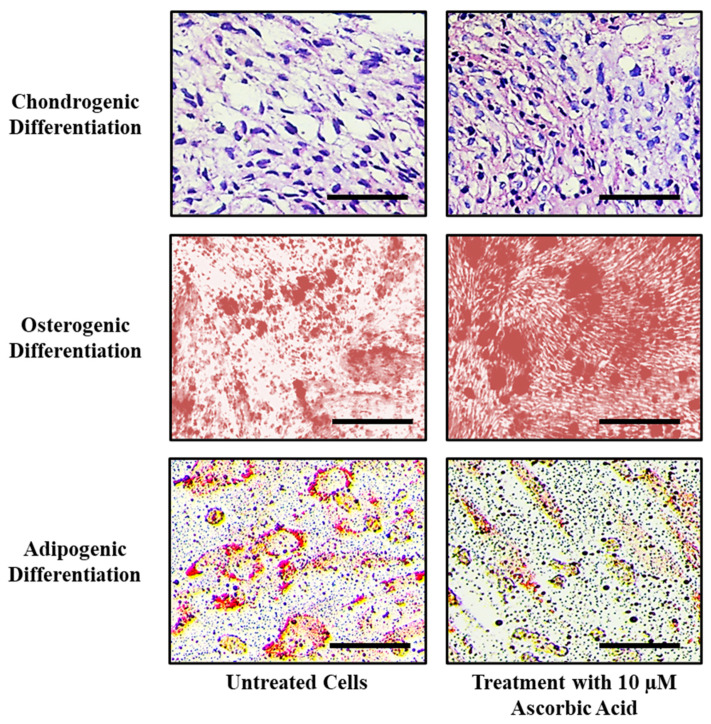
Comparative differentiation of SHEDs into chondrocytes, osteoblasts, and adipocytes after ascorbic acid treatment. Scale bar = 100 µm.

Treatment with ascorbic acid enhanced chondrogenic and osteogenic differentiation, as observed by highly stained cells in treated groups. Ascorbic acid reduced the adipogenic differentiation potential, as evidenced by fewer oil droplets formation (Figure 2).

### 3.5. Ascorbic Acid Treatment Augments Growth Factors and Cytokines Secretion by SHEDs

**Figure 3:**
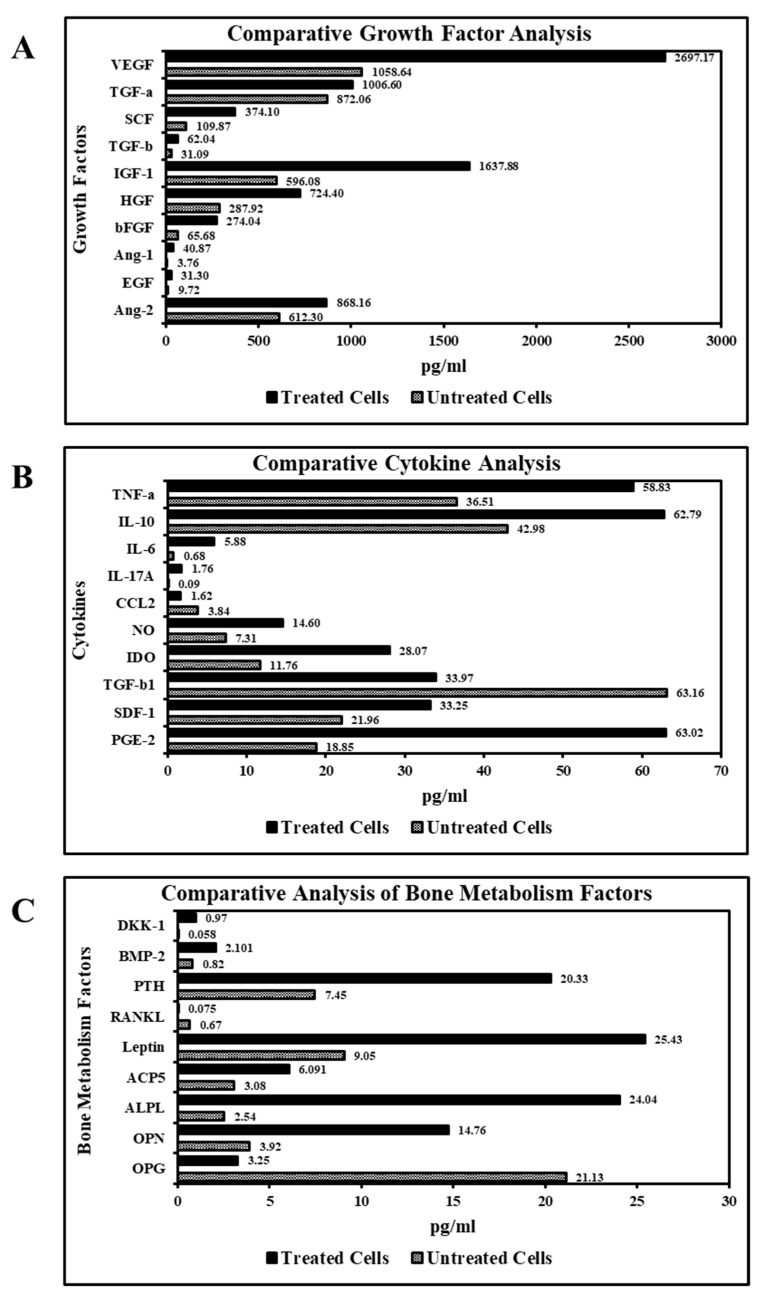
Analysis of secretome of SHEDs for growth factors, cytokines, and bone metabolism factors analysis. (**A**). Comparative analysis of growth factors in SHED-secretome before and after treatment with ascorbic acid. (**B**). Comparative analysis of cytokines in SHED-secretome before and after treatment with ascorbic acid. (**C**). Comparative analysis of bone metabolism factors in SHED-secretome before and after treatment with ascorbic acid.

Ascorbic acid treatment resulted in an increased secretion of growth factors necessary for the tissue regeneration and homeostases, such as VEGF, SCF, IGF-1, HGF, bFGF, Ang-1, and EGF. Treatment with ascorbic acid increased anti-inflammatory cytokines, such as NO, IDO, PGE-2, IL-10, and IL-6. Ascorbic acid treatment resulted in a reduction in the secretion of inflammatory cytokines CCL2, TGF-b1 in comparison with untreated cells (Figure 3A,B). Ascorbic acid treatment of the SHEDs enhanced the secretion of a diverse panel of bone metabolism factors responsible for the development and growth of the bone, such as PTH, ALPL, and OPN (Figure 3C).

### 3.6. Ascorbic Acid Treatment Boosts the Expression of Stemness-Related Genes

**Figure 4:**
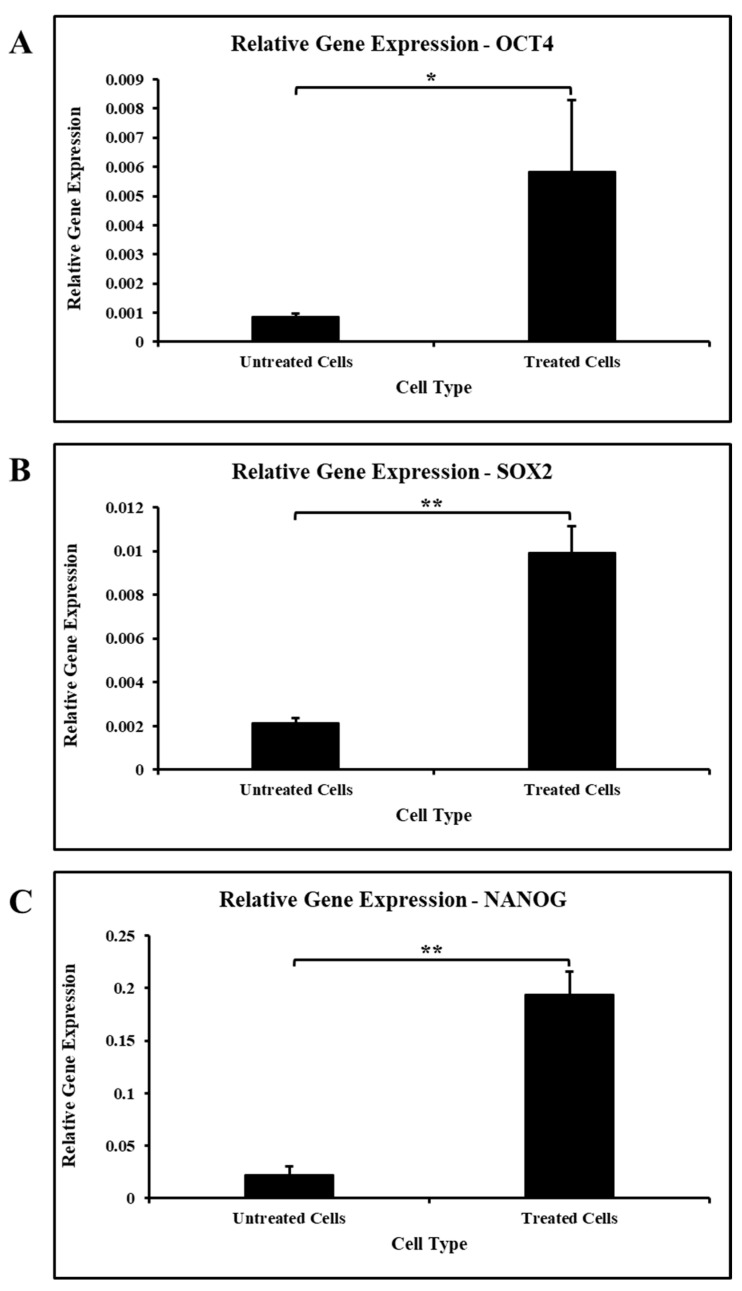
Gene expression analysis of stemness-related genes in SHEDs. (**A**). Comparative gene expression analysis of OCT4 in SHEDs before and after treatment with ascorbic acid. (**B**). Comparative gene expression analysis of SOX2 in SHEDs before and after treatment with ascorbic acid. (**C**). Comparative gene expression analysis of NANOG in SHEDs before and after treatment with ascorbic acid. * *p* < 0.05, ** *p* < 0.001.

On treatment with ascorbic acid, cells demonstrated an increased expression of stemness-related genes, such as OCT4, SOX2, and NANOG, in comparison with untreated cells (Figure 4A–C).

## 4. Discussion

Stem cells are pluripotent nature in nature, have plasticity, and are unlikely to cause adverse reactions. Due to these attributes, stem cell therapy has been considered for the management of various diseases [4]. However, a major concern is the source of the stem cells and the microenvironment that influences their properties. An ideal source of MSCs would be one that was easily accessible through non-invasive or minimally invasive procedures. An ideal MSC source should contain a good number of stem cells and prove easy for isolation and culture without chemical or mechanical stresses. The pulp from a tooth satisfies all these criteria and is a viable tissue to acquire cells of clinical significance [2,4,8]. However, it is important to note that therapeutic extraction of healthy teeth is performed only during specific orthodontic therapy or extraction of wisdom teeth. Therefore, procurement of healthy pulp tissue from permanent teeth may not be possible for all patients. Dental pulp from deciduous teeth would be an ideal source of stem cells as the procedure is minimally invasive and uncomplicated. Human teeth contain specific areas termed stem cell niches that regulate the participation of dental pulp stem cells in homeostasis, tissue repair, and regeneration [46]. 

The DPSCs located in several niches are associated with the nerves and vessels of the central region, cell-rich zone, and outer layer. Specific interactions with the local microenvironment in these niches and stem cells regulate mitosis and differentiation via signaling pathways, such as Notch, WNT/β-Catenin signaling, growth factors, i.e., vascular endothelial growth factor, transforming growth factor (TGF)-β and ECM [47]. The stem cells obtained from the dental pulp of deciduous teeth have several advantages, such as greater propagation rate, enhanced clonogenicity, and elevated osteoinductive capacity, in comparison with other types of MSCs [4,28]. Recently there are several studies have reported the role of natural compounds that aid in tissue healing and repair. Inchingolo et al. have reported the tissue healing role of Bromelain in causing a reduction in postoperative edemas, following extraction of third molars [48]. 

In this regard, ascorbic acid (AsA), commonly known as vitamin C, can modify the cell culture microenvironment. AsA is an antioxidant and anti-inflammatory agent. It has been widely used as an additive in the culture medium for cell growth and differentiation. However, its effect on dental pulp stem cells obtained from deciduous teeth has not been researched. This study examines the effect of ascorbic acid on SHED.

The results revealed that SHEDs expressed CD73, CD90, and CD105, which are mesenchymal stem cell markers (Figure 1B). The expression of CD34 and CD45 (both hematopoietic lineage markers) and HLA-DR (MHC class-II cell surface receptor) was found to be negative for all SHEDs cultures (Figure 1B). These results show that SHEDs are free of other lineage cells, making them appropriate for allogeneic clinical transplantation use. We observed that the SHEDs could exhibit tri-lineage differentiation. These results are congruent with the ideal culture conditions of MSCs as demonstrated by the positive expression of surface markers, the constant potential for proliferation, proper telomerase activity, and the persistent aptitude to differentiate into particular lineages [1,16,23].

Treatment of stem cells with ascorbic acid reduced the adipogenic differentiation potential and enhanced chondrogenic and osteogenic differentiation. This may be due to the vital role ascorbic acid plays in the process of bone and cartilage development. Ascorbic acid deficiency can lead to decreased chondrocyte proliferation, impaired matrix synthesis, and a reduction in the number of osteoblasts [49,50]. Temu et al. reported similar results in ATDC5 cells where ascorbic acid was found to promote chondrocyte differentiation by facilitating collagenous matrix formation. This matrix formation was found to mediate activation of the ERK signaling pathway, thereby promoting differentiation [51]. The chondrocyte differentiation of SHED should be further examined for the management of cartilage disorders, such as rheumatoid arthritis, developmental disorders like achondroplasias, chondrodysplasia, and osteoarthritis, and orthopedic treatments.

One of the key characteristics of stem cells is paracrine secretion [10,24,52]. Stem cells secrete key molecules for the repair and regeneration of the tissues [14,31]. In the present study, we observed that SHEDs could secrete a plethora of growth factors and cytokines. Following treatment of MSCs with ascorbic acid, we observed an increased secretion of the growth factors necessary for tissue regeneration and homeostasis. There was an increased secretion of anti-inflammatory cytokines, while the secretion of inflammatory cytokines declined. YL Wu et al. reported similar findings in human skin fibroblasts treated with ascorbic acid showing an increased expression of hepatocyte growth factors. Our results echo findings by EH Kong et al., who reported that ascorbic acid increased anti-inflammatory cytokines and decreased expression of pro-inflammatory cytokines in mouse splenocytes [53,54]. These results could provide a basis for research into clinical applications, such as induction of regeneration following injury and immunomodulation [1,55].

The SHEDs were found to secrete a diverse panel of bone metabolism factors. Treatment with ascorbic acid enhanced the secretion for most of the factors involved in the development and growth of the bone tissue. KM Choi et al. reported similar findings on the effect of ascorbic acid on bone marrow-derived mesenchymal stem cell proliferation and differentiation [56]. This could be explained by the fact that ascorbic acid is essential for expressing osteoblastic markers and mineralization [57]. Ishikawa et al. reported that ascorbic acid-induced upregulation of type I collagen production; α2β1 integrin (a major receptor of type I collagen) and alkaline phosphatase activity on periodontal ligament cells on the early osteoblastic differentiation of periodontal ligament cells [58]. The osteoblast differentiation of stem cells has potential clinical applications in enhancing implant osseointegration and sinus augmentation [2,3,4,59,60,61,62].

The stemness properties of MSCs must be preserved for a long duration. This preservation of stemness could be achieved through expressing stemness and pluripotency maintaining genes [15,52,63]. In our study, ascorbic acid treatment increased the expression of the OCT4, SOX2, and NANOG. Yu et al. reported a similar effect of ascorbic acid on adipocyte-derived stem cells. They reported that A2-P was found to enhance the stemness of adipocyte-derived stem cells through collagen synthesis via the ERK1/2 pathway and cell sheet formation. They also found ASC transdifferentiation capabilities into the neuron and hepatocyte-like cells, endothelial and epidermal lineages under various induction conditions [64]. Wahyuningsih et al. established that ascorbic acid increased mitosis and decreased the aging of adipocyte-derived stem cells [5]. Thus, further research focused on the enhanced proliferation and stemness of ascorbic acid-treated stem cells could have implications in tissue regeneration. This study is limited by its in vitro design and is preliminary. Studies on the molecular pathways through which ascorbic acid exerts effects on SHEDs combined with long-term in vivo studies will provide insights into potential clinical applications.

## 5. Conclusions

Ascorbic acid (vitamin C) at lower concentrations increased chondrogenesis and osteogenesis, while decreasing the adipogenesis in SHEDs. Treatment with ascorbic acid enhanced the secretion of growth factors, anti-inflammatory cytokines, and factors related to bone metabolism. Ascorbic acid-treated SHEDs may provide newer treatment strategies for the regeneration of teeth and associated structures. It has potential applications in pulp capping, apexogenesis, management of cleft lip, and other congenital anomalies in pediatric patients.

## Figures and Tables

**Table 1:** List of primers.

Gene	Forward Primer	Reverse Primer
NANOG	5′-TTT GTG GGC CTG AAG AAA ACT-3′	5′-AGG GCT GTC CTG AAT AAG CAG-3′
OCT4	5′-GTG GAG GAA GCT GAC AAC AA-3′	5′-ATT CTC CAG GTT GCC TCT CA-3′
SOX2	5′-CCA GCA GAC TTC ACA TGT CC-3′	5′-ACA TGT GTG AGA GGG GCA GT-3′
ACTIN	5′-AGA GCT ACG AGC TGC CTG AC-3′	5′-AGC ACT GTG TTG GCG TAC AG-3′
